# 

**Published:** 1875-02

**Authors:** 


### Books and Pamphlets Received.

Compendium of Children's Diseases. A Handbook for Practitioners and Students. By Johann Steiner, M. D. Translated from the second German Edition by Lawson Tait, F.R.C.S. New York: D. Appleton & Co., 1875. Buffalo: Herger & Ulbrich.

A Practical Treatise on the Medical and Surgical Uses of Electricity. Including Localized and General Faradization; Localized and Central Galvanization; Electrolysis and Galvano-Cautery. By Geo. M. Beard, A. M., M. D., and H. D. Rockwell, A. M., M. D. Second Edition. New York: Wm. Wood & Co., 1875. Buffalo: H. H. Otis.

The Histology and Histochemistry of Man. A Treatise on the Elements of Composition and Structure of the Human Body. By Heinrich Frey, Prof. of Medicine in Zurich. Translated from the Fourth German Edition. By Arthur E. J. Barker, M. D. New York: D. Appleton & Co., 1875. Buffalo: Herger & Ulbrich.

Dental Pathology and Surgery. By S. James H. Salter, M. D., F.R.S. New York: Wm. Wood & Co., 1875. Buffalo: H. H. Otis.

Pulmonary Tuberculosis: Its Pathology, Nature, Symptoms, Diagnosis, Causes, Hygiene and Medical Treatment. By Addison P. Dutcher, M. D. Philadelphia: J. B. Lippincott & Co., 1875. Buffalo: Martin Taylor.

Eating for Strength. By M. L. Holbrook, M. D., aided by numerous competent assistants. New York: Wood & Holbrook, 1875.

On the Treatment of Pleurisy. By John W. Corson, M. D. New York: Wm. Wood & Co., 1875.

A Series of American Clinical Lectures, edited by E. C. Seguin, M. D. Vol. I, No. I. On Diseases of the Hip Joint, by Lewis H. Sayre, M. D. New York: G. P. Putnam's Sons, 1875.

Transactions of the American Ophthalmological Society. Tenth Annual Meeting, Newport, July 1874. New York: Wm. Wood & Co., 1874.

Annual Address before the Philadelphia County Medical Society. By Washington L. Atlee, A. M., retiring President. February 1875.

Scleritis Syphilitica: Its Pathology Course and Treatment. By Fred R. Sturgis, M. D. From Archives of Dermatology, Vol. I, No. 2.

On the Relations of the Neruous System to Diseases of the Skin. By L. Duncan Bulkley, A. M., M. D. From Archives of Electrology and Neurology. November, 1874.

Insects of the Pond and Steam. By A. S. Packard, Jr. Boston: Estes & Lauriat, 1875.

### Medical and Surgical Journal

PUBLISHED MONTHLY,

Containing Original Articles, Reports of Medical Societies and Hospitals, Editorial, Reviews, Correspondence, Army News, etc., etc; including the usual variety of Medical Periodical Publications. Specimen copies sent on application.

Address Buffalo Medical & Surgical Journal, Buffalo, N. Y.

TERMS OF SUBSCRIPTION, - - $3.00 PER YEAR, IN ADVANCE.

